# *In Vitro* Detection of Acaricidal Resistance Status of* Rhipicephalus (Boophilus) microplus* against Commercial Preparation of Deltamethrin, Flumethrin, and Fipronil from North Gujarat, India

**DOI:** 10.1155/2015/506586

**Published:** 2015-12-14

**Authors:** K. P. Shyma, Jay Prakash Gupta, Veer Singh, K. K. Patel

**Affiliations:** ^1^Department of Veterinary Parasitology, Sardarkrushinagar Dantiwada Agricultural University, Sardarkrushinagar 385506, India; ^2^Department of Animal Genetics & Breeding, Sardarkrushinagar Dantiwada Agricultural University, Sardarkrushinagar 385506, India; ^3^Banas Dairy, Palanpur, Banaskantha, Gujarat 385001, India

## Abstract

*Rhipicephalus (Boophilus) microplus* is the most common tick species in India infesting cattle and buffaloes and causing significant economic losses to dairy and leather industries by adversely affecting the milk production and quality of hides. A study to evaluate the acaricide resistance status of* Rhipicephalus (Boophilus) microplus *to deltamethrin, flumethrin, and fipronil was conducted on the samples collected from organized and unorganized farms of North Gujarat state, where treatment failures were reported frequently. Adult Immersion Test (AIT) and Larval Packet Test (LPT) were conducted using field strain for determination of 50 and 95% lethal concentration of deltamethrin, flumethrin, and fipronil. Results obtained by the Adult Immersion Test showed low grade resistance (level I, RF > 5) has been developed against both deltamethrin and fipronil. However, deltamethrin by performing Larval Packet Test showed moderate grade resistance (level II, RF > 25). Larval packet performed by flumethrin also revealed low grade resistance, level I. The data on field status of acaricide resistance from the area with diversified animal genetic resources will be helpful to adopt suitable strategy to overcome the process of development of resistance in ticks.

## 1. Introduction

*Rhipicephalus (Boophilus) microplus* (Canestrini, 1888) is a widely prevalent tick and assumes great significance in a tropical country like India, where the warm, humid climate favours its perpetuation and propagation. The cost caused by tick and tick borne diseases has not been systematically studied in India but roughly it has been estimated in the tune of 2000 crore rupees per annum [1]. Northern Gujarat is western most subtropical part located in Gujarat plains and hills agroclimatic zone of Indian subcontinent, where* R. (B). microplus* is the most prevalent tick species found to infest cattle [2]. Chemical control has been the main strategy to overcome tick infestations but repeated application of these chemicals leads to the development of resistance in the ticks and the selection of resistant ticks is considered as the main hindrance for successful pest and vector control program [3]. A number of nonorganophosphate (OP) classes of pesticides have been developed which are effective against arthropod pests, environmentally safe, and relatively less toxic to mammals and other nontarget organisms when compared to OP compounds. Among these pesticides, the synthetic pyrethroids, deltamethrin and flumethrin, are commercially available in India and presently the predominant acaricides used to control tick in the country [4, 5]. There are reports indicating development of various grade of resistance against pyrethroids in field strains [3, 5–7]. Besides this, fipronil is a part of new generation of products used to combat invertebrates [8] and has been recently introduced in the Indian market. Only recently, resistance against chemical acaricides has been detected in both one-host and multihost ticks collected from North Gujarat [9, 10]. Recently resistance status of fipronil was diagnosed by [11–13] but there are very few reports on this from India to date [14]. Although livestock keepers from the field have often reported treatment inefficiencies or failure of these chemicals, there is absence of published report of resistance status of these acaricides from this part of the country. Keeping all these in view, it was quite demanding to assess the resistance status of commonly used acaricides from the area which contributes substantially to the livestock wealth as well as livestock products of the country.

## 2. Materials and Methods

### 2.1. Acaricides

Farmers of the areas under study have reported frequent applications of chemical acaricides in particular deltamethrin, flumethrin, and to lesser extent fipronil, without maintaining an optimum concentration for the control of ticks mainly due to low efficacy of most of the marketed products. For dose dependent bioassay, commercially available preparations of deltamethrin (1.25%) and flumethrin (1%) were diluted in distilled water whereas fipronil (0.25%) was diluted in 25% acetone to make different concentrations, namely, 30, 35, 50, 70, and 105 ppm for deltamethrin, 50, 100, 200, 400, and 600 ppm for flumethrin, and 8, 10, 12, 16, 18, and 24 ppm for fipronil.

### 2.2. Collection and Preparation of Field Isolates of* Rhipicephalus (Boophilus) microplus* Ticks

Areas with report of high incidence of tick infestation were selected in the present study. There was reports of frequent applications of commonly available acaricides particularly deltamethrin by the farmers without maintaining an optimum concentration for the control of ticks, mainly due to low efficacy of most of the marketed products. Veterinarians of the locality often complain about treatment failure in tick infestation case.

The fully engorged adult* R. (B.) microplus* ticks were collected from different organized and unorganized farms of North Gujarat from where there was history of frequent treatment failure. North Gujarat is predominated with the dairy farms comprising mainly crossbred cattle population. In organized farms, the animals are kept in zero grazing system whereas unorganized farms are mainly possessed by small farmers in villages. Ticks were handpicked from the body of cattle of varying age and from the vicinity of their pens. The ticks were washed in tap water and dried on an absorbent paper. A total of 285 adult engorged female ticks were used for the present study. Out of this, 15 ticks were separated and were held individually at 28°C and 75–85% relative humidity in labeled glass bottle with the mouth covered by muslin cloth for oviposition. The eggs were allowed to hatch to larvae in 18–25 days under similar conditions of incubation. The larvae were used for performing “Larval Packet Test” (LPT). The remaining 270 ticks were gathered into eighteen groups each of 15 ticks (one for each concentration of chemical acaricides and two as a control; one for deltamethrin and flumethrin and another for fipronil). Out of 15 ticks in each group, 3 replicates were used to estimate the acaricidal effects of respective concentration of chemical acaricide by AIT.

### 2.3. Adult Immersion Test

The Adult Immersion Test (AIT) was conducted as per the protocol described by Drummond et al. [15]. The ticks were immersed in 10 mL of different concentrations of chemical acaricides, namely, 30, 35, 50, 70, and 105 ppm for deltamethrin, 50, 100, 200, 400, and 600 ppm for flumethrin, and 8, 10, 12, 16, 18, and 24 ppm for fipronil, for two minutes in a 25 mL beaker with gentle agitation. The control group for deltamethrin and flumethrin was immersed in water whereas for fipronil it was immersed in acetone. The ticks were then placed on Petri dishes over Whatman filter paper number 1. All the Petri dishes with treated ticks were kept at room temperature for 24 h. After 24 h, ticks were transferred to glass vials covered with muslin cloth and kept in desiccators having 75–85% relative humidity and placed in BOD incubator at 28°C. These ticks were observed for oviposition and death up to 15 days. Egg mass was observed under the same incubation conditions in a BOD incubator for the next 30 days. The percentage of adult tick mortality and the weight of the eggs laid by the treated ticks were recorded in comparison with the control. The eggs were incubated at the same condition and the percentage of hatched eggs was estimated visually. The index of egg laying and percentage inhibition of fecundity were calculated using the following formulae (1) and (2), respectively [16, 17]:

(1) Reproductive  Index  RI=Weight  of  eggs  laid  (mg)Weight  of  adult  females  mg

(2) Percentage  inhibition  of  oviposition  IO  %=RIcontrol  group−RItreated  groupRIcontrol  group×100.

### 2.4. Larval Packet Test (LPT)

The LPT was performed on 12- to 14-day-old larvae as prescribed by FAO [16] with some minor modifications. For each active ingredient, a dilution series was set up the same as was used for AIT in order to obtain a concentration gradient resulting in 0 to 100% larval mortality. For each dilution series, a negative control was used. For each concentration, five replicates were made. A volume of approximately 0.6 mL of each solution was applied to Whatman number 1 (3.75 × 8.5 cm) filter paper. After saturation, the compound was dried by keeping the filter paper for 30 minutes in incubator at 37°C. Treated and dried parallelograms of paper were folded in half forming equilateral triangular packets and sealed on the sides with adhesive tapes forming an open ended packet. After insertion of approximately 100 larvae, the open side of each packet was sealed with adhesive tape and the packets were placed in a desiccators kept in BOD incubator maintained at 28°C and 75–85% RH. After 24 hours, the packets were opened, and both the live and the dead larvae were counted. The ability of the larvae to walk on the surface of the filter paper was used as the criterion for determining whether larvae were dead or alive. The dose response data of all the three acaricides using reference susceptible lines were analyzed.

### 2.5. Statistical Analysis

All the data were expressed as mean ± SE. Groups were compared using one-way analysis of variance using Graph Pad Prism 4 software. A value of* P* < 0.05 was considered as statistically significant.

### 2.6. Probit Analysis

Dose response data were analyzed by probit method [18]. The 50% (LC_50_) and 95% (LC_95_) lethal concentrations of deltamethrin, flumethrin, and fipronil against* R. (B.) microplus* were determined by applying regression equation analysis to the probit-transformed data of mortality. Using LC_50_ values of reference lines, resistance factor (RF) was worked out by the formula given by Castro-Janer et al. [12]:

(3) Resistant  factor  RF=LC50  value of field ticksLC50  value of susceptiple ticks.

For calculation of RF of deltamethrin and fipronil, data on reference tick line IVRI-I was used [3, 14, 19]; however resistance factor of flumethrin was not calculated as reference tick line is yet to be developed. On the basis of RF, the resistance status in the field population of* R. (B.) microplus* was classified as susceptible (RF < 1.4), level I resistance (1.5 < RF < 10.0), level II resistance (10.1 < RF < 25.0), level III resistance (26 < RF < 40), and level IV resistance (RF > 41).

## 3. Results

### 3.1. Deltamethrin and Flumethrin Resistance Status

**Figure 1:**
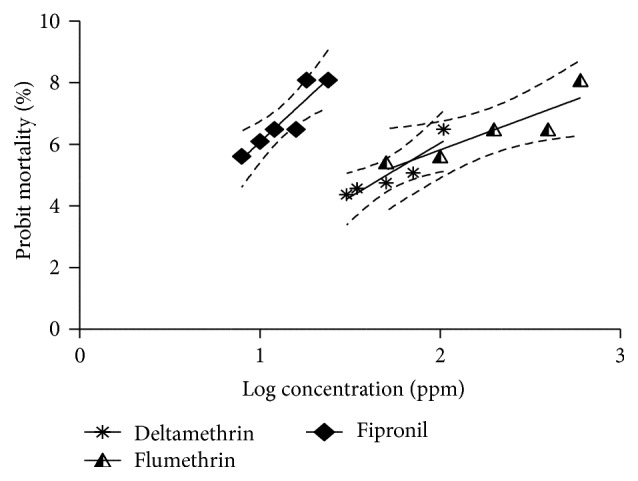
Comparative probit mortality in fully engorged adult* R. (B.) microplus* subjected to dose response AIT assay with deltamethrin, flumethrin, and fipronil.

**Figure 2:**
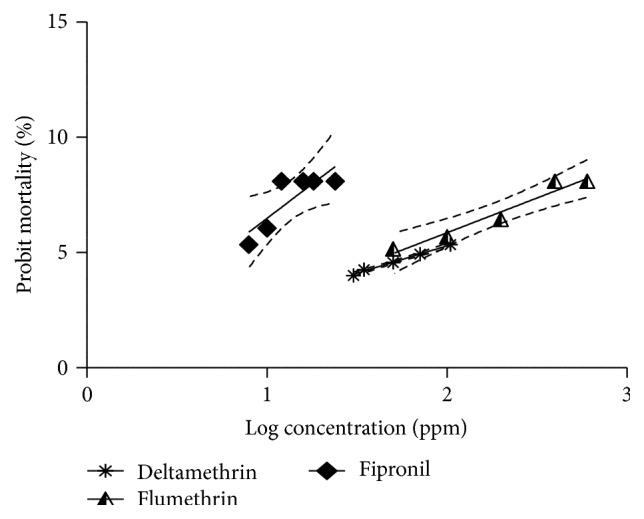
Comparative probit mortality in larvae of* R. (B.) microplus* subjected to dose response LPT assay with deltamethrin, flumethrin, and fipronil.

**Figure 3:**
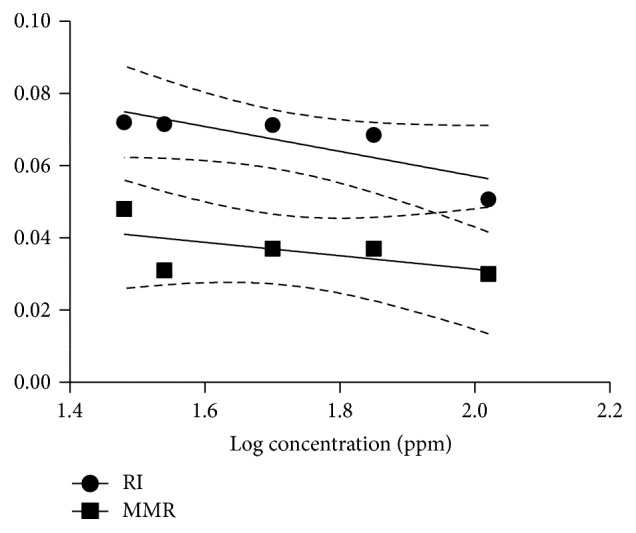
Reproductive index and mean egg mass per replicate in fully engorged adult* R. (B.) microplus* subjected to dose response AIT assay with deltamethrin.

**Figure 4:**
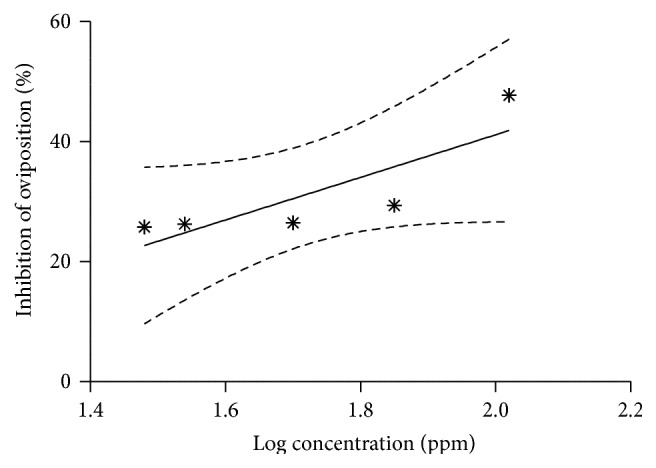
Inhibition of oviposition (IO %) in fully engorged adult* R. (B.) microplus* subjected to dose response AIT assay with deltamethrin.

The dose dependent response of both Adult Immersion Test (AIT) and Larval Packet Test (LPT) after using different concentrations of deltamethrin and flumethrin is shown in Table 1. The narrow confidence intervals in LC_50_ and LC_95_ values of deltamethrin and flumethrin presented in Table 2 affirm the homogeneity of samples collected. When AIT was performed, the RF value of* R. (B.) microplus* for deltamethrin was recorded as 3.76, a level I resistance status, whereas RF, when LPT was conducted, was recorded as 6.38, a level II resistance status, indicating that LPT is more sensitive than AIT; this was in accordance with the findings of Kumar et al. [14]. This may be due to the fact that LPT utilizes relatively much higher number of larvae (minimizing error) when compared with the AIT. The regression graphs of adult and larval probit mortality of* R. (B.) microplus* plotted against log values of progressively increasing concentrations of both pyrethroids along with fipronil are shown in Figures 1 and 2, respectively. In comparison to deltamethrin, the resistance to flumethrin was comparatively low and appears that it adversely affected the reproductive physiology of the treated ticks, more significantly at higher doses. The LC_50_ and LC_95_ values determined for flumethrin were 51.83 and 181.50 ppm. The coefficients of determination (*R* ^2^ values) of estimations were more than 80%, indicating good fitting of the data in the probit model (Table 2). The high slope values recorded for deltamethrin indicate a high drug response in concentration gradient manner on tick biology. Mean% adult mortality within 15 days (MA15), mean eggs mass per replicate (MMR), reproductive index (RI), and inhibition of oviposition (IO) of ticks were found to be significantly inhibited by deltamethrin in dose dependent manner; however flumethrin completely occludes the egg laying at all concentrations. The slope of MMR and RI of ticks treated with deltamethrin is shown in Figure 3 whereas slope of IO % is shown in Figure 4. These slopes were not significantly different from zero and recorded as −0.034, −0.019, and 35.51, respectively, for RI, MMR, and IO with coefficient of determination (*R* ^2^) of around 70%.

### 3.2. Fipronil Resistance Status

Fipronil produces dose dependent mortality in both adults and larva and causes complete death of both at higher dosage (Table 1). The slope values calculated for fipronil were higher when compared with the pyrethroids. The complete mortality of larva is occurring at concentration of 20 ppm or more while adults died completely at only 50 ppm or above concentrations.* R. (B.) microplus* presents level I resistance status against fipronil both by LPT and by AIT and the RF calculated was 2.36 and 3.78, respectively (Table 2).

## 4. Discussion

The work was undertaken based on the reports of treatment failure in the region and people were looking for alternate to the chemical acaricides [20]. There was report of indiscriminate use of deltamethrin and introduction of flumethrin and fipronil in the recent years. Selection for acaricide resistance in tick populations is a major consequence of using chemical acaricides and is the principal threat to the efficacy of synthetic pyrethroids, particularly deltamethrin, for the control of ticks. The purpose of the present study was an analysis of deltamethrin, flumethrin, and fipronil resistance status in field samples of* R. (B.) microplus* in North Gujarat, India.

The present data demonstrate the comprehensive information on the level of resistance in* R. (B.) microplus* to commonly used synthetic pyrethroids and fipronil using bioassays, AIT, and LPT. The results revealed comparatively higher level of resistance against deltamethrin but gradual development of resistance to fipronil in* R. (B.) microplus*. The study revealed comparatively high resistant factor (RF) of 6.38 for LPT against deltamethrin which was ranked as level II resistance. Sharma et al. [5] also reported highest level of resistance against deltamethrin in* R. (B.) microplus* from neighboring agroclimatic zone (western dry region). Flumethrin and fipronil caused complete occlusion of oviposition. Flumethrin seems to be comparatively effective against* R. (B.) microplus* mainly due to its late introduction in the Indian market. Further, farmers were reluctant to adopt this chemical owing to longer milk withholding period. Mendes et al. [21] reported LC_50_ of susceptible (Mozo) strain as 50 ppm by LPT. Resistance factor of ticks against flumethrin could not be determined owing to lack of data on reference tick line; however, resistance to synthetic pyrethroids is usually described in terms of family resistance where ticks simultaneously develop resistance to more than one compound of the group [19, 22]. Data on reference tick lines for flumethrin were, however, available for other countries, but Kumar et al. [14] opined that country specific discriminating concentration for different acaricides is a mandatory requirement to monitor level of resistance in ticks as there are many factors contributing to the development of resistance such as geographical location, climate, economic status of the farmers, dose and frequency of acaricides application, and breed of animals.

The slope values of field ticks, for both AIT and LPT, were comparatively lower than laboratory susceptible tick, suggestive of presence of heterogeneous population of ticks in the field having both resistant and susceptible alleles in the populations that allow the presence of homozygous and heterozygous individuals [13]. The slopes of MMR and RI of engorged females exposed to various concentrations of deltamethrin were negative, thus indicating that although the increase in concentration of the drug could not cause mortality in all the exposed ticks, the egg laying capacity or the efficacy of conversion of live weight into egg mass decreased among the surviving females.

The present data demonstrate the first report of low level resistance against fipronil in* R. (B.) microplus* of Gujarat, by both AIT and LPT method. The standard bioassay promoted by FAO for testing resistance to acaricides in* R. (B.) microplus* is the Larval Packet Test (LPT) originally described by Stone and Haydock [23]. However, AIT has also been used successfully for resistance monitoring in different countries [5, 6, 12, 14, 24, 25]. This study further affirms the suitability of AIT as we could detect higher slope value in the field isolates. Both AIT and LPT were found to be suitable tools for characterization of acaricide resistance in field tick isolates. In comparison to AIT, LPT requires 3–6 weeks more time which may prove to be a long time when dealing with resistance outbreak. However, this test can be conducted even with a few ticks available in the field and multiple tests can be carried out with thousands of larvae produced by a few engorged female ticks [14], whereas AIT can be conducted with ease and data can be generated within 2 weeks' time.

The results of the current study advocates that relatively higher concentration of flumethrin and fipronil and much higher concentration of deltamethrin would be required for causing significant mortality, thus indicating that the dose at which these acaricides are being used in field conditions is becoming ineffective and needs to be revalidated. Further, in the current study, commercially available acaricides were used to assess the efficacy of these widely used drugs which could not have been possible with the use of analytical grade acaricides as commercial products are prepared with many proprietary ingredients and it is difficult to assess the responses due to individual components of the formulations [26]. Based on the data obtained on the emerging problem of resistance in* Rhipicephalus (B.) microplus* to chemical acaricides which are recently being used heavily in the region, an alert on good practices aiming at tick control is required to be recommended in order to monitor resistance and judicious use of acaricides.

## Figures and Tables

**Table 1:** Dose dependent response of Adult Immersion Test (AIT) and Larval Packet Test (LPT) to various commercial preparations against *R. (B.) microplus*.

Concentration (ppm)	ATR ± SE	MA15 ± SE	MMR ± SE	RI ± SE	IO (%)	Hatching (%) (visual)	MDL ± SE (%)
Deltamethrin							
Control	0.56 ± 0.02	06.67 ± 06.67	0.049 ± 0.004	0.0970 ± 0.010	00.00	100	00.8 ± 0.37
30	0.64 ± 0.02	26.67 ± 06.67	0.048 ± 0.004^*∗*^	0.0720 ± 0.007	25.77	80	16.2 ± 1.07^*∗∗∗*^
35	0.52 ± 0.01	33.33 ± 06.67	0.031 ± 0.003^*∗*^	0.0715 ± 0.003	26.28	80	23.0 ± 0.83^*∗∗∗*^
50	0.53 ± 0.02	40.00 ± 14.53	0.037 ± 0.003^*∗*^	0.0713 ± 0.005	26.49	80	33.8 ± 0.66^*∗∗∗*^
70	0.53 ± 0.01	53.33 ± 06.67^*∗*^	0.037 ± 0.002^*∗*^	0.0685 ± 0.006^*∗*^	29.38	80	47.8 ± 0.92^*∗∗∗*^
105	0.58 ± 0.01	93.33 ± 06.67^*∗∗∗*^	0.030 ± 0.003^*∗∗∗*^	0.0507 ± 0.005^*∗∗*^	47.73	80	63.6 ± 0.51^*∗∗∗*^

Flumethrin							
50	0.51 ± 0.01	66.67 ± 06.67^*∗∗∗*^	0.000 ± 0.000	0.000 ± 0.000	00.00	0	56.4 ± 0.93^*∗∗∗*^
100	0.62 ± 0.03	73.33 ± 13.33^*∗∗∗*^	0.000 ± 0.000	0.000 ± 0.000	00.00	0	75.6 ± 1.29^*∗∗∗*^
200	0.58 ± 0.02	93.33 ± 06.67^*∗∗∗*^	0.000 ± 0.000	0.000 ± 0.000	00.00	0	92.82 ± 1.24^*∗∗∗*^
400	0.63 ± 0.02	93.33 ± 06.67^*∗∗∗*^	0.000 ± 0.000	0.000 ± 0.000	00.00	0	100.0 ± 0.00^*∗∗∗*^
600	0.51 ± 0.01	100.00 ± 00.00^*∗∗∗*^	0.000 ± 0.000	0.000 ± 0.000	00.00	0	100.0 ± 0.00^*∗∗∗*^

Fipronil							
Control	0.51 ± 0.01	00.00 ± 00.00	0.050 ± 0.001	0.098 ± 0.002	00.00	100	01.2 ± 0.37
8	0.57 ± 0.02	73.33 ± 06.67^*∗∗∗*^	0.000 ± 0.000	0.000 ± 0.000	00.00	0	63.4 ± 0.68^*∗∗∗*^
10	0.58 ± 0.02	86.66 ± 06.67^*∗∗∗*^	0.000 ± 0.000	0.000 ± 0.000	00.00	0	85.8 ± 1.28^*∗∗∗*^
12	0.56 ± 0.02	93.33 ± 06.67^*∗∗∗*^	0.000 ± 0.000	0.000 ± 0.000	00.00	0	100.0 ± 0.00^*∗∗∗*^
16	0.50 ± 0.01	93.33 ± 06.67^*∗∗∗*^	0.000 ± 0.000	0.000 ± 0.000	00.00	0	100.0 ± 0.00^*∗∗∗*^
18	0.54 ± 0.02	100.00 ± 00.00^*∗∗∗*^	0.000 ± 0.000	0.000 ± 0.000	00.00	0	100.0 ± 0.00^*∗∗∗*^
24	0.57 ± 0.01	100.00 ± 00.00^*∗∗∗*^	0.000 ± 0.000	0.000 ± 0.000	00.00	0	100.0 ± 0.00

ATR: average tick weight per replicate; SE: standard error; MA15: mean% adult mortality within 15 days; MMR: mean eggs mass per replicate; RI: reproductive index; IO (%): percent inhibition of oviposition; MDL (%): mean dead larva

^*∗*^ *P* < 0.001; ^*∗∗*^ *P* < 0.01; ^*∗∗∗*^ *P* < 0.05.

**Table 2:** Mortality slope, LC_50_ and LC_95_ confidence limit, resistance factor, and resistance level against deltamethrin, flumethrin, and fipronil as determined by AIT and LPT of ticks collected from North Gujarat, India.

Variable	Slope ± SE (95% CL)	*R* ^2^ (%)	LC_50_ (95% CL)	LC_95_ (95% CL)	RF^a^	RL^b^
Adult Immersion Test						
Deltamethrin	3.51 ± 0.86 (0.78–6.23)	84.00	50.35 (46.29–59.34)	147.11 (120.94–230.89)	3.76	I
Flumethrin^*∗*^	2.17 ± 0.60 (0.25–4.10)	81.00	41.81 (36.40–54.54)	239.20 (173.85–498.26)		
Fipronil	5.43 ± 1.15 (2.24–8.61)	85.00	6.36 (6.02–7.07)	12.76 (11.23–17.10)	3.78	I

Larval Packet Test						
Deltamethrin	2.38 ± 0.10 (2.06–2.70)	99.50	75.24 (66.35–95.83)	367.74 (275.10–716.91)	6.38	II
Flumethrin^*∗*^	3.01 ± 0.40 (1.74–4.29)	94.90	51.83 (46.93–62.74)	181.5 (144.31–307.53)		
Fipronil	5.94 ± 1.94 (0.55–11.33)	69.99	5.66 (5.38–6.23)	10.65 (9.49–13.91)	2.36	I

^*∗*^Resistance factors and levels could not be detected as baseline data is yet to be developed.

^a^RF: LC_50_ of field populations/LC_50_ of susceptible population.

^b^Susceptible = RF < 1.4; level I = 1.5 < RF < 5; level II = 5.1 < RF < 25; level III = 26 < RF < 40; level IV = RF > 41; S = susceptible.
